# Effects of Concurrent Aerobic Plus Resistance Training on Blood Pressure Variability and Blood Pressure Values in Patients with Hypertension and Coronary Artery Disease: Gender-Related Differences

**DOI:** 10.3390/jcdd9060172

**Published:** 2022-05-27

**Authors:** Giuseppe Caminiti, Marco Alfonso Perrone, Maurizio Volterrani, Ferdinando Iellamo, Giuseppe Marazzi, Serena Selli, Alessio Franchini, Elvira Padua

**Affiliations:** 1 Cardiology Rehabilitation Unit, S. Raffaele IRCCS, 00163 Rome, Italy; maurizio.volterrani@sanraffaele.it (M.V.); iellamo@uniroma2.it (F.I.); giuseppe.marazzi@sanraffaele.it (G.M.); serena.selli@libero.it (S.S.); franchini.fkt@gmail.com (A.F.); 2 Department of Human Science and Promotion of Quality of Life, San Raffaele Open University, 00163 Rome, Italy; elvira.padua@uniroma5.it; 3 Department of Clinical Sciences and Translational Medicine, University of Rome Tor Vergata, 00133 Rome, Italy; marco.perrone@uniroma2.it

**Keywords:** concurrent exercise: blood pressure variability, hypertension

## Abstract

The purpose of this study was to compare changes in blood pressure variability (BPV) and blood pressure (BP) values occurring in response to concurrent training (CT) between the two genders. A total of 35 males and 20 women aged 55–80 years, with hypertension and coronary artery disease, were included. They underwent a 12-week CT program. The aerobic component of CT was performed according to the rate of perceived exertion while the intensity of the resistance component was set at 60% of 1 repetition maximum for the first 4 weeks and then increased to 80%. BP and BPV were evaluated at baseline and at the end of the CT program through 24 h ambulatory blood pressure monitoring. After 12-weeks, 24 h and daytime systolic BPV decreased in both men and women without significant between-groups differences. Twenty-four-hour daytime and nighttime diastolic BPV decreased in both genders with a significantly greater decrease in women compared to men. Twenty-four-hour daytime systolic and 24 h diastolic BP decreased in men while they were unchanged in women. In conclusion, CT induced similar reductions of systolic BPV in men and women and a greater decrease in diastolic BPV in women. Conversely, CT decreased BP values in males but not in females. CT appears to be an effective intervention for reducing BPV in both genders.

## 1. Introduction

Hypertension is the most common comorbidity encountered in patients with coronary artery disease (CAD) undergoing secondary prevention programs. Despite available data underlining the importance of obtaining an optimal blood pressure (BP) control in such high-risk patients, it has been estimated that this goal is reached in just over half of them [1]. Aside from BP values, an elevated BP variability (BPV) is growingly recognized as a consistent risk factor associated with target organs damage [2] and increased cardiovascular events independently from hypertension [3,4]. Up to now, there are few data about effective pharmacological and non-pharmacological treatments for lowering BPV [5]. Exercise training, in particular aerobic exercise, is a recognized non-pharmacological intervention for lowering BP in hypertensive subjects, and for containing BP raise in pre-hypertension [6]. Conversely, its role in reducing BPV has not been established yet. Concurrent training (CT) consisting of the addition of resistance exercises to aerobic exercise during the same session appears to be particularly suitable for managing cardiovascular risk factors in elderly subjects since it has demonstrated simultaneous benefits in the cardiorespiratory fitness, metabolic profile, and muscle endurance and strength [7,8]. Overall, current literature shows a positive impact of CT in lowering BP in several populations. In a recent meta-analysis, CT showed similar long-term anti-hypertensive effects then aerobic continuous exercise [9]. In elderly male patients with hypertension and CAD, CT elicited similar acute post-exercise hypotension to aerobic training alone [10]. A 12-week CT decreased BP and improved arterial stiffness in hypertensive elderly women as well [11] and prevented cardiac and renal oxidative stress together with BP reduction in ovariectomized rats [12,13]. Regarding the effects of CT on BPV, available data are not large enough for making conclusive statements, especially considering that published studies have shown contradictory results [14,15]. In male patients with hypertension and CAD, when compared with aerobic continuous training and high-intensity interval training, CT was the most effective modality for reducing BPV both after a single session and after 12-weeks of exercise training [15,16]. Conversely, two recent small studies enrolling women with and without hypertension found no effects of CT on BPV [17,18]. Notably in both studies, the intensity of the resistance component of CT was set at 60% of 1 repetition maximum (1-RM) equal to that used in studies involving men and BPV response to higher intensities has not been investigated yet. Moreover, we did not find studies comparing the BPV response to CT between the two genders. Therefore, in the present study, we evaluated whether a 12-week CT with resistance exercise performed at increasing intensity (60% of 1-RM for the first four weeks and then 80% for the remaining 8-weeks) would be effective in reducing BPV in both genders. The primary endpoint of this study was to compare changes in 24 h systolic BPV between men and women with hypertension and underlying CAD, following a 12-week CT program. Secondary endpoints were changes on 24 h diastolic BPV and 24 h systolic BP.

## 2. Materials and Methods

### 2.1. Population

The study included a total of 55 patients, 35 males and 20 females, evaluated for being enrolled in a cardiac rehabilitation program at the cardiac rehabilitation centre of S. Raffaele IRCCS, Rome. The following inclusion criteria were adopted: diagnosis of hypertension; underlying coronary artery disease; patients being in stable clinical conditions and stable pharmacological therapy (without changes occurring in the last three months); sedentary lifestyle: patients not being enrolled in exercise training programs in the previous six months; age between 55 and 80 years. Exclusion criteria were: BP levels exceeding 160/100 mmHg; significant heart valve diseases; hypertrophic cardiomyopathy; signs and/or symptoms of myocardial ischemia during a preliminary ergometric test; uncontrolled arrhythmia; neurological and/or orthopedic conditions contraindicating or limiting exercises; significant chronic obstructive pulmonary disease (FEV1 < 50%); symptomatic peripheral arterial disease. Moreover, patients were excluded if they changed their pharmacological treatment during the study period.

### 2.2. Study Protocol

The study was conducted as a prospective, longitudinal single-center open pilot study, with two arms corresponding to the two genders. It was preliminarily approved by the ethical committee of S. Raffaele IRCCS, Rome (prot *n* = 25/20). All patients gave informed consent to participate in the study, which conformed to the principles outlined in the Declaration of Helsinki. Patients meeting the inclusion/exclusion criteria, after signing informed consent, underwent a baseline evaluation including clinical and pharmacological history, assessment of body mass index, and waist circumference. On the same day, patients performed an ergometric symptom-limited test in order to rule out exercise-induced ischemia and ventricular arrhythmias during exercise. The ergometric test was performed on a treadmill (Mortara Instr, Bologna, Italy) and a standard Bruce protocol was adopted for each patient. At each stage of the test, RPE was assessed through the Borg 6–20 scale. On the same day, patients had an adaptive session during which they familiarized themselves with the gym equipment and the established exercise modality. At the end of this adaptive session, patients underwent the assessment of 1-repetition maximum (1-RM) for the different muscle groups involved in the resistance phase of the training. Within ten days from the first visit, all subjects started an exercise training program lasting 12-weeks and including three sessions every week. Within a week from the first visit and up to three days before the first planned exercise session, all subjects underwent 24 h ambulatory blood pressure monitoring (ABPM). Within a week from the last session, they were re-evaluated and performed another ergometric test and 24 h ABPM. The effects of the exercise training protocol on functional capacity were also evaluated by the six minute walk test (6MWT) [19] and the rate of perceived exertion (RPE) was assessed through the Borg’s 6–20 scale [20]. Both tests were performed at baseline and within a week from the last exercise session.

### 2.3. 24 h ABPM

ABPM was performed with a validated oscillometric device (BP one, London, UK), with the BP cuff placed on the non-dominant arm. The recording was programmed to obtain BP readings at 15 min intervals from 6.00 a.m. to 10.00 p.m. and 20 min intervals from 10.00 p.m. to 6.00 a.m. BP readings considered for data analysis included only those obtained from 8:00 a.m. to 8:00 p.m. (daytime) and from midnight to 6:00 a.m. (nighttime) for excluding transition periods from daytime and nighttime. On the day of the ABPM, patients were instructed to maintain their usual activities and medications. All ABPMs were performed on working days. The majority of our patients were retired workers and were engaged in low-intensity activities during the working days in which they performed ABPM (e.g., shopping, walking, etc.). Twelve out of forty (30%) patients were still active workers at the time of the study protocol. All of them were office employers and were not engaged in physically demanding jobs. In the days of ABPM, they spent 6–8 h at work, mostly seated behind a desk. Data from ABPM were accepted if at least 75% of the measurements were obtained successfully. The following parameters were considered: 24 h systolic BP, daytime systolic BP, nighttime systolic BP, 24 h diastolic BP, daytime diastolic BP, nighttime diastolic BP, and their respective time-related variability (BPV). BPV has been evaluated by means of average real variability (ARV), according to the formula reported by Mena et al. [21]. Standard deviation (SD) for all reported variables was also evaluated, as a metric of BPV for comparison.

### 2.4. Exercise Training Protocol

Exercise training sessions were performed in the gym of our hospital. Patients performed aerobic exercises before resistance exercises in each session. Each session lasted 60 min and was preceded by 10 min of warm-up and followed by 10 min of cool-down. Exercise sessions were planned as follows: 40 min of aerobic training on a treadmill; 20 min of resistance training (Technogym Wellness System, Technogym, Cesena, Italy). The intensity of the aerobic component of the whole study training was established by means of the RPE method with an intensity target of 13–14 during the whole study. Regarding the resistance component of the sessions, it consisted of the following exercises: leg press and extension, shoulder press, chest press, low row, and vertical traction. The intensity of each resistance exercise was established through the assessment of the corresponding 1-RM: for each exercise, patients performed a warm-up set (8–0 repetitions) at 50–60% of their perceived 1-RM. Then they were asked to perform one repetition at their maximal effort. This last movement was carried out three times, with 2–3 min rest between efforts, and the highest value of strength registered was used as 1-RM [22]. For each exercise, the intensity was set at 60% of 1-RM during the first month and was then increased to 80% of 1-RM for the last 2 months of the training program. The 1-RM test was assessed at baseline and then it was repeated every two weeks. The following muscle groups were involved: quadriceps, back muscles, deltoids, and biceps. Patients performed two sets for every exercise; each set included 10 repetitions per set. Patients had 2 min of rest between sets. Each session was preceded by 10 min of warm-up and followed by 10 min of cool-down. The exercise sessions were supervised by a rehabilitation team including three physiotherapists that were involved in the following tasks: helping patients to set up treadmills and dynamometers at each session, and checking that exercises were carried out correctly by the patients. A cardiologist with experience in the cardiac rehabilitation field and a nurse were also supervising the exercise sessions. Patients’ heart rhythm was monitored by telemetry in the initial sessions, for safety reasons.

### 2.5. Statistical Analysis

By considering the lack of previous studies confronting exercise-induced changes in BPV between the two genders we did not assess a sample size. Data are expressed as mean ± SD. The assumption of normality was checked using the Shapiro–Wilks hypothesis test. Pre- and post-exercise data of normally-distributed variables were assessed using a repeated measure two-way ANOVA, with Bonferroni corrections for post hoc testing. Not normally-distributed variables were assessed using the Kruskal–Wallis test and Bonferroni corrections for post hoc testing. The level of significance was set at *p* < 0.05. Data were analyzed using SPSS software (version 20.0 IBM Corp, Amonk, New York, NY, USA).

## 3. Results

Participants were matched regarding age, BMI, the prevalence of previous myocardial infarction, and the number of antihypertensive drugs taken (Table 1). Overall, 52 patients completed the protocol and were included in the analyses. Two patients (one in the male and one in the female group) dropped out before completion for their unwillingness to continue the exercise program. One further patient of the male group was excluded from analyses due to protocol violation in terms of changes in the antihypertensive medications. All subjects completed the exercise sessions without symptoms, and the quality of their ABPM recorded was considered satisfactory. There was high compliance with the exercise protocols in both groups (attended sessions/planned sessions × 100: males = 89.4%; females = 86.1%). At the end of the study, the duration of ergometric test increased significantly in both groups (men from 413.2 ± 44 s to 482.3 ± 71 s, *p* = 0.0001, women from 366.5 ± 46 s to 432.3 ± 54 s, *p* = 0.0011); without significant between-groups difference (*p* = 0.11). Distance at 6MWT increased significantly in both groups (men from 395.6 ± 65 m to 468.1 ± 68 m, *p* = 0.0036; women from 342.8 ± 44 m to 447.5 ± 51 m, *p* = 0.0015); without significant between-groups difference (*p* = 0.07). The Borg’s scale score decreased from 10.9 ± 2.2 to 7.3 ± 1.9 in males and from 11.5 ± 2.7 to 7.9 ± 2.4 in females between-groups, *p* = 0.36). BMI did not change in comparison to baseline values in both groups (men from 29.5 ± 6.3 kg/m^2^, to 29.1 ± 7.3 kg/m^2^,; women from 28.9 ± 8.3.1 kg/m^2^, to 28.41 ± 5.3 kg/m^2^, *p* = 0.32 for between-groups comparison.

### 3.1. Blood Pressure Variability

Changes in BPV are summarized in Table 2. According to ARV: Twenty-four hour (men = −0.7 ± 0.3; women = −1.1 ± 0.6; *p* 0.14) and daytime (men = −0.8 ± 0.5; women = −1.9 ± 0.8; *p* 0.26) systolic BPV decreased in both genders without significant between-groups differences (between groups, *p* 0.13). Nighttime systolic BPV was unchanged in both groups. Twenty-four hour (men = −0.5 ± 0.3; women = −2.8 ± 1.1; *p* 0.023), daytime (men = −0.5 ± 0.2; women = −3.8 ± 1.7; *p* 0.001) and nighttime (men = −0.6 ± 0.4; women = −1.8 ± 0.4; *p* 0.003) diastolic BPV decreased in both groups, but to a greater extent in the female group. According to SD: similarly to ARV, we observed reductions of BPV at 12-weeks in both males and females; with significant between groups differences regarding diastolic BPV.

### 3.2. Blood Pressure Values

Twenty-four hour and daytime systolic BP decreased in men (−5.1 ± 1.2 and −5.7 ± 1.6, respectively) while they were unchanged in women (between groups, *p* 0.008 and *p* 0.003, respectively). Nighttime systolic BP decreased (−4.4 ± 1.9) in men and presented a non-significant increase in women (+1.4 ± 0.4), (between groups, *p* 0.0001). Twenty-four hour and daytime diastolic BP decreased in men (−4.4 ± 0.8 and −4.1 ± 1.3, respectively) and women (−1.5 ± 0.4 and −2.1 ± 0.6 respectively) without significant between-groups differences. Nighttime diastolic BP decreased in men (−3.8 ± 1.7) while it was unchanged in women (between groups *p* 0.02).

## 4. Discussion

The most important finding of the present study is that a CT program lasting 12-weeks effectively reduced short-term BPV in both males and women patients with hypertension and underlying CAD with a significantly greater decrease in diastolic BPV in women compared to males. To our knowledge, this is the first study showing a positive impact of an exercise training modality on short-term BPV in women. This result appears to have potential implications in the clinical practice: an elevated BPV has been associated with an increased risk of cardiovascular events in patients with already diagnosed CAD [23,24]. Regarding diastolic short-term BPV, its prognostic role has also been underlined recently: in a study involving more than 9000 subjects with hypertension, diastolic short-term BPV was directly and independently related to cardiovascular mortality across all ages [25]. Despite our results needing further confirmations in larger studies, they extend to women CT benefits previously described only in men with CAD [15,16] and suggest a potential role for CT in the treatment of both systolic and diastolic BPV in women. The CT-mediated reduction of systolic and diastolic BPV in males observed in the present study represents a confirmation of previous studies published by our group [15,16]. It should be noted however that in the present study we used a step-up approach regarding the resistance component of CT that was performed at 60% of 1-RM for the first month and then increased at 80% for the remaining two-thirds of the exercise training program. Therefore, we can hypothesize that the beneficial effects of CT on BPV in males are maintained when the intensity of the resistance component of CT is increased. Regarding the female gender, our results differ from those obtained by two recent small studies conducted on a female with or without hypertension: in the study of Mariano et al. [18] BPV did not change after CT; in another study, BPV decreased acutely after a single session of CT but not after a training period lasting ten weeks [17]. Again, differences between studies might depend on different training intensities adopted: in the present study women, like men, performed the resistance component of the training in a step-up fashion (from 60% to 80% of 1-RM) while in the other studies, the resistance component of CT was constantly performed at 60% of 1-RM. Moreover, in the study of Matias et al. [17], the 1-RM was assessed at baseline and re-evaluated after five weeks, at the middle point of their exercise program. In our study, 1-RM was assessed every two weeks; it is possible that a more frequent evaluation of the 1-RM test might have helped to train our patients as close as possible to the established intensities avoiding undertraining. An additional explanation could be the longer duration of our exercise protocol (12 weeks versus 10 weeks in the previous studies on women). Overall our data suggest that a sustained effect of CT on BPV in women appears when the intensity of the resistance component of the training is increased. Undoubtedly, data on the female gender are still scant and the sample size needs to be implemented: in particular further studies exploring the BPV response to different exercise intensities are needed in order to confirm our results and to explain the heterogeneity of the current literature. At the end of the training program, we observed a significant reduction of both systolic and diastolic BP values in males, while they did not change in females. The observed result in men complies with recent studies that used similar exercise intensities [26,27]. With regard to the female gender, the present study agrees with current literature showing that CT reduced only slightly BP in post-menopausal women. In a recent meta-analysis evaluating the effects of CT on BP of post-menopausal women, Xi et al. [28] showed that CT reduced systolic BP by 0.81 mmHg and DBP by 0.62 mmHg. Several potential variables might have affected the lack of BP reduction in women after CT in the present study; among them are the training intensity and frequency. In studies that documented BP reductions in women after CT, the intensity of the resistance component of CT was set at 60% of 1-RM and the greater intensity used in our study might have caused an excessive sympathetic activation in women. Regarding the weekly frequency of CT sessions, our patients performed CT three times a week: this might have affected negatively the BP response in women since, a frequency of two sessions/week is associated with a greater hypotensive response in women, as suggested by a subgroup analysis of the meta-analysis performed by Xi et al. [28]. Limitations. The most important limitation of this study is the small sample size; since we did not find previous research evaluating gender differences in the BPV response to CT, we conceived the present research as a preliminary study; further larger studies on this topic are needed in order to clarify the effects of exercise training in BPV in patients with cardiovascular disease. The study lacks a control group (without exercise) and therefore, it is not possible to attribute a robust effect of the concurrent exercise on BPV. The age of the study population ranged from 55 and 80 years and it is possible that the BPV response to CT may have been different across the age spectrum; however, the small sample size of this study prevented us from performing a sub-analysis according to the age. Despite all components of the rehabilitation team of this study being experts in cardiac rehabilitation programs, we did not include a physical and rehabilitation medicine specialist that is a professional figure expected in rehabilitation teams [29]. Our results should be restricted to hypertensive patients with already diagnosed cardiovascular disease and cannot be generalized to other populations.

## 5. Conclusions

In this preliminary study, we observed that a 12-week CT program was effective in reducing BPV in both genders while it reduced BP values only in men. We think that our results go in the direction of more individualized treatment of hypertension and high BPV in patients with cardiovascular diseases. However, considering the preliminary nature of our data they need to be confirmed in other larger studies.

## Figures and Tables

**Table 1:** Antropometric and clinical features of the population.

	Males (*n* = 34)	Females (*n* = 18)
Age, years	71.4 ± 11.5	70.3 ± 14.5
BMI, kg/m^2^	29.5 ± 6.3	28.9 ± 8.3
Waist circumference, mm	104 ± 13.1	91 ± 10.7
Previous AMI	22 (63)	13 (65)
Previous PCI/CABG	23/12	12/8
Comorbidities		
Hypertension, *n* (%)	35 (100)	20 (100)
Diabetes, *n* (%)	8 (22)	4 (20)
Hypercholesterolemia, *n* (%)	25 (71)	16 (80)
Previous smoke habit, *n* (%)	21 (60)	13 (65)
Treatment		
ACE-Is/ARBs	29 (83)	18 (90)
Betablockers	24 (68)	13 (65)
CCA	16 (46)	10 (50)
Diuretics	11 (31)	8 (40)
Statins	32 (91)	20 (100)

AMI = acute myocardial infarction; PCI = percutaneous coronary intervention; CABG = coronary artery bypass grafting; ACE-I = angiotensin converting enzyme inhibitors; ARBs = angiotensin receptor blockers; CCA = calcium-channel antagonists.

**Table 2:** Changes in blood pressure variability, blood pressure values, and heart rate in males and females.

	Men		Women	
	*Baseline*	12-weeks	*Baseline*	12-weeks
BPV-ARV-mmHg				
24 h SBPV	8.6 ± 2.5	7.9 ± 2.2 *	10.4 ± 3.0	9.3 ± 1.8 *
Daytime SPBV	8.4 ± 2.9	7.6 ± 1.8	10.7 ± 3.4	8.8 ± 2.6 *
Nighttime SBPV	9.2 ± 2.1	8.7 ± 2.5	9,8 ± 2.2	10.5 ± 2.9
24 h DBPV	6.6 ± 1.5	6.1 ± 1.9 *	9.4 ± 2.7	6.6 ± 1.6 *^,^^†^
Daytimel DBPV	6.5 ± 2.2	6.0 ± 2.1	10.2 ± 1.7	6.4 ± 1.9 *^,^^†^
Nighttime DBPV	7.0 ± 1.7	6.4 ± 1.5 *	8.7 ± 2.,1	6.9 ± 1.5 *^,^^†^
BPV,-SD-mmHg				
24 h SBPV	11.1 ± 2.4	10.6 ± 1.8	12.7 ± 3.6	13.3 ± 2.0
Daytime SPBV	13.2 ± 3.1	12.8 ± 1.8	13.1 ± 2.3	12.1 ± 2.6
Nighttime SBPV	11.2 ± 2.1	11.5 ± 2.5	11.1 ± 2.7	11.5 ± 2.5
24 h DBPV	7.2 ± 1.8	6.9 ± 1.2	11.9 ± 2.7	9.6 ± 1.4 *^,^^†^
Daytime DBPV	9.8 ± 2.4	8.6 ± 2.1 *	10.7 ± 1.7	8.0 ± 1.9 *^,^^†^
Nighttime DBPV	8.6 ± 1.7	7.7 ± 1.5 *	9.9 ± 2.,1	8.5 ± 1.5 *^,^^†^
BP values, mmHg				
24 h SBP,	120.1 ± 12.6	115.0 ± 22.5 *^,^^†^	115.4 ± 15,7	115.7 ± 18.3
Daytime SPB	126.1 ± 12.6	120.4 ± 20.3 *^,^^†^	120.1 ± 12,6	120.3 ± 24.4
Nighttime SBP	113.2 ± 15.3	108.8 ± 14.8 *^,^^†^	110.6 ± 20.2	112.0 ± 16.2
24 h DBP	71.5 ± 12.6	67.6 ± 19.6 *	69.7 ± 11.7	68.2 ± 6,1
Daytime DPB	76.5 ± 11.0	72.4 ± 17.7 *	74.6 ± 10.1	72.5 ± 12.4 *
Nighttime DBP	66.2 ± 12.4	62.4 ± 14.1 *^,^^†^	64.9 ± 11.3	64.3 ± 10.5
HR, bpm				
24 h	61.6 ± 13.7	61.0 ± 16.7	63.9 ± 14.0	63.1 ± 15.3
Daytime	62.4 ± 14.1	61.8 ± 12.4	63.9 ± 14.9	63.2 ± 14.5
Nighttime	59.3 ± 17.0	56.7 ± 15.8 *	59.3 ± 13.7	57.2 ± 16.1 *

BPV = blood pressure variability; SBPV = systolic blood pressure variability; DBPV = diastolic blood pressure variability; SBP = systolic blood pressure; DBP = diastolic blood pressure; HR = heart rate. * *p* < 0.05 baseline vs. 12 weeks. ^†^ *p* < 0.05 between groups.

## Data Availability

The data presented in this study are available on request from the corresponding author.
